# Clinical Prediction of High-Turnover Bone Disease After Kidney Transplantation

**DOI:** 10.1007/s00223-021-00917-1

**Published:** 2021-10-19

**Authors:** Satu M. Keronen, Leena A. L. Martola, Patrik Finne, Inari S. Burton, Xiaoyu F. Tong, Heikki P. Kröger, Eero O. Honkanen

**Affiliations:** 1 Abdominal Center, Department of Nephrology, University of Helsinki and Helsinki University Hospital, (Haartmaninkatu 4), P.O. Box 372, 00029 HUS, Finland; 2 Kuopio Musculoskeletal Research Unit (KMRU), University of Eastern Finland, P.O.Box 1627, 70211 Kuopio, Finland; 3 Department of Orthopedics, Traumatology, and Hand Surgery, Kuopio University Hospital, P.O.Box 100, 70029 KYS, Finland

**Keywords:** Kidney transplantation, CKD-MBD, Hyperparathyroidism

## Abstract

Bone histomorphometric analysis is the most accurate method for the evaluation of bone turnover, but non-invasive tools are also required. We studied whether bone biomarkers can predict high bone turnover determined by bone histomorphometry after kidney transplantation. We retrospectively evaluated the results of bone biopsy specimens obtained from kidney transplant recipients due to the clinical suspicion of high bone turnover between 2000 and 2015. Bone biomarkers were acquired concurrently. Of 813 kidney transplant recipients, 154 (19%) biopsies were taken at a median of 28 (interquartile range, 18–70) months after engraftment. Of 114 patients included in the statistical analysis, 80 (70%) presented with high bone turnover. Normal or low bone turnover was detected in 34 patients (30%). For discriminating high bone turnover from non-high, alkaline phosphatase, parathyroid hormone, and ionized calcium had the areas under the receiver operating characteristic curve (AUCs) of 0.704, 0.661, and 0.619, respectively. The combination of these markers performed better with an AUC of 0.775. The positive predictive value for high turnover at a predicted probability cutoff of 90% was 95% while the negative predictive value was 35%. This study concurs with previous observations that hyperparathyroidism with or without hypercalcemia does not necessarily imply high bone turnover in kidney transplant recipients. The prediction of high bone turnover can be improved by considering alkaline phosphatase levels, as presented in the logistic regression model. If bone biopsy is not readily available, this model may serve as clinically available tool in recognizing high turnover after engraftment.

**Supplementary Information:**

The online version contains supplementary material available at 10.1007/s00223-021-00917-1.

## Introduction

Advances in immunosuppressive medication after kidney transplantation have notably enhanced the short-term survival rates of kidney allografts [1]. Subsequently, the primary target in the management of kidney transplant recipients nowadays is the improvement of long-term health and quality of life. Although successful kidney transplantation restores kidney function in the majority of the patients, the disordered mineral metabolism due to kidney disease often reverses only partially [2–16]. Post-transplantation bone disease comprises persistent hyperparathyroidism, bone loss, and osteonecrosis. It results primarily from continuing or evolving pre-existing mineral and bone disease, further aggravated by poor allograft function [17, 18]. In observational studies, post-transplantation bone disease, especially persistent hyperparathyroidism, has been linked to an increased risk of graft loss, exacerbation of cardiovascular disease, and death [9, 15, 17, 19].

The incidence of fractures in kidney transplant recipients is markedly high compared with the general population [20–26]. Besides the decreased quality of life, fractures in kidney transplant recipients have been associated with an increased risk of hospitalization and mortality [18]. Both the catabolic effect of persistent hyperparathyroidism and the use of glucocorticoids have been suggested as contributors to bone loss after kidney transplantation [17, 27, 28]. The quantity and quality of bone are impaired also by factors independent of kidney transplantation (e.g., dialysis vintage, sex, age, hypogonadism, and physical inactivity) [17, 18, 27].

The histomorphometric analysis of iliac bone biopsy has been selected as the gold standard in the assessment of bone metabolism [29]. However, the invasive nature of this procedure and the lack of specific histopathological expertise required for the sample analysis hinder its widespread use. Accordingly, the number of bone biopsy studies in kidney transplant recipients is scarce [2, 4, 5, 8, 13, 16, 30]. Although some bone biomarkers have shown promising utility in the differentiation of bone turnover, their potential to replace bone histomorphometry in patients with the post-transplant bone disease is still limited [27].

We conducted this retrospective study to investigate the role of bone biopsy in the evaluation of patients with persistent hyperparathyroidism after kidney transplantation. Another aim was to evaluate, whether clinical and biochemical data could help to recognize high-turnover bone disease.

## Methods

After obtaining approval from the Research Ethics Board of the Division of Medicine, Helsinki University Central Hospital (approval no. 413/13/03/00/09) and the Institutional Review Board of the Hospital District of Helsinki and Uusimaa (HUS/33/2010, HUS/269/2017, and HUS/333/2019) with the waiver of informed consent for medical record review, we retrospectively screened the medical records of kidney transplant recipients referred for bone biopsy between January 1, 2000 and December 31, 2015 due to the suspicion of high-turnover bone disease. Bone biopsy was considered indicated to verify the diagnosis and to exclude low bone turnover before therapeutic interventions, especially parathyroidectomy. Patients with estimated glomerular filtration rate (eGFR) exceeding 30 ml/min/1.73 m^2^ and persistently elevated intact parathyroid hormone (PTH) with or without hypercalcemia at the time of biopsy were included. Elevated PTH level was determined according to the valid PTH assessment at the time of biopsy (>47–73 ng/L). Only bone biopsies with the assessment of bone turnover were included and patients with repeat biopsies were included only once.

There have been some changes in clinical practice during the 15 years period while bone biopsies were collected. In 2004, cinacalcet was introduced for the treatment of secondary hyperparathyroidism. However, due to the lack of official indication and imbursement for post-transplant hyperparathyroidism, the use of cinacalcet has not been widely adopted for the treatment of persistent hyperparathyroidism after kidney transplantation at our institution. The immunosuppressant tacrolimus was introduced in Finland in 2001 and its use has gradually increased since that time. Concomitantly the use of corticosteroids almost halved between 1998 and 2008.

### Data Collection

Electronic patient charts were reviewed for demographics (age, sex, medical comorbidities, and medications at the time of biopsy) and laboratory findings. We also collected information on parathyroidectomies performed before and after kidney transplantation.

The following laboratory results at the time of or within four months preceding the bone biopsy were recorded: plasma inorganic phosphate and ionized calcium (iCa), alkaline phosphatase (ALP), PTH, plasma creatinine, and eGFR (measured by the Chronic Kidney Disease Epidemiology Collaboration equation) [31]. Routine methods were used to analyze phosphate and iCa. Levels of ALP were measured by enzyme-linked immunosorbent assay (Roche Modular). The reference range was 60–275 U/L until April 28, 2004 and since April 29, 2004 35–105 U/L. Intact PTH levels were studied by using an immunoradiometric assay (LIAISON, reference range 15–60 ng/L) between May 15, 2000 and September 9, 2001, by immunochemiluminometric assay (Siemens IMMULITE 2000, reference range 8–73 ng/L) from September 10, 2001 to May 31, 2011, and since June 1, 2011 by electrochemiluminescence immunoassay (Roche Modular, reference range 12–47 ng/L). Since January 15, 2014, the reference range for the same method was changed to 15–65 ng/L.

### Bone Biopsy and Histomorphometric Analysis

Iliac crest biopsies were obtained after local anesthesia using an electric drill (Straumann, Switzerland) until the year 2005 and thereafter by using vertical technique and 8G–11G needle (T-Lok, Angiotech, Reading, PA, USA) 5–14 days after the second labeling with tetracycline (500 mg three times/day over two separate two-day periods with a 10-day interlabel time).

The method for quantitative histomorphometry has been described earlier [32]. Histomorphometric analyses were performed at standardized sites in cancellous bone at ×200 magnification using a semiautomatic image analyzer [Osteoplan II system (Carl Zeiss, Thornwood, NY, USA) until the year 2004 and thereafter BioquantOsteoII, (Bioquant Image Analysis Corporation, Nashville, TN, USA)].

Bone turnover was assessed by the bone formation rate per unit of bone surface (BFR/BS, normal reference value 18–38 µm^3^/µm^2^/year) and activation frequency (Ac. F, normal reference value 0.49–0.72/year) [33]. In the complete absence of tetracycline labeling or if only one label was found in the cancellous bone area the assessment of bone turnover was made using osteoblastic (Ob.S/BS, %) and osteoclastic surfaces (Oc.S/BS, %). Rehman`s age matched reference values > 2 SD or < -2 SD were used to define high turnover and low turnover, respectively [34]. Abnormal mineralization was identified when osteoid surface/bone surface (OS/BS, %) was more than ± 2 SD compared with the mean value [33] and/or mineralization lag time (Mlt, days) was over 100 days [35]. The normal range of cancellous bone volume/tissue volume (BV/TV) was 16.8–22.9% [34].

The final classification of bone turnover, however, was not based merely on bone histomorphometric parameters (BFR/BS, Ac. F), but on the consensus statement of two experienced histomorphometrists (HK, IB) also.

### Statistical Analysis

For statistical analysis, we divided bone biopsy findings into three groups according to bone turnover (low, normal, and high). Due to the small number of patients with low bone turnover, we combined this group with the normal bone turnover group. Mineralization and bone volume were determined according to turnover-mineralization-volume classification [33]. To allow comparisons between PTH values at different time points, we used the conversion equation y(IMMULITE2000) = 0.99×(LIAISON)-0.6 (R = 0.98, *n* = 103 with plasma samples) and y(Modular) = 0.52× (IMMULITE2000) + 11 ng/L (reference range determined by comparison of samples). To allow comparisons between ALP values at different time points, we converted levels of ALP taken between January 1, 2000 and April 28, 2004 by using the conversion equation y = ALP (old) ×0.48. We imputed 9 ALP values using a k-nearest neighbor approach [36]. The variables used for imputation were sex, age, the time between transplantation date to bone biopsy, dialysis vintage, previous parathyroidectomy, bone turnover, and the levels of iCa and PTH. In 13 patients with only plasma total calcium level available, we converted levels of total calcium to iCa by multiplying with 0.52. To compare differences in parameters between turnover groups, we used Mann–Whitney U-test and Chi-square test for continuous and categorical variables, respectively. Kendall’s tau correlation coefficient was applied to determine correlations between continuous variables [37].

We constructed logistic regression equations to estimate whether the various variables could independently predict high bone turnover in the bone biopsy. Variables that showed a significant (*p* < 0.1) association with high bone turnover in the univariable logistic regression analysis were further examined in multivariable logistic regression analysis (variables evaluated: age, diabetes, eGFR, smoking, the timing of bone biopsy after engraftment, cumulative dose of glucocorticoids, previous parathyroidectomy, prevalent and incident fractures, dialysis vintage, and PTH, iCa, Pi, ALP and the use of bisphosphonates at the time of bone biopsy). PTH was evaluated as linear and as transformations (logarithmic and square root). The logarithmic transformation was chosen as it gave the lowest deviance (−2 log-likelihood) of the logistic regression model. The five most significant covariates were the use of bisphosphonates, iCa, ALP, natural logarithmic PTH (lnPTH), and the timing of biopsy after engraftment. When P-value removal was set to <0.10 in backward conditional analysis, the selected variables were iCa, ALP, and ln PTH. We performed all analyses with SPSS for Windows (version 25, SPSS, Chicago, IL, USA), and all values are presented as the median and interquartile range (IQR, 25–75 percentiles). Statistical significance was defined as a two-sided P-value lower than 0.05. For internal validation, we formed 1000 bootstrap samples AUCs for each sample using the caret package in R (R Foundation for Statistical Computing). To compare the difference in AUC values between the prediction model and covariates we used DeLong`s test for two correlated ROC curves [38].

## Results

### Characteristics of Kidney Transplant Recipients

**Fig. 1:**
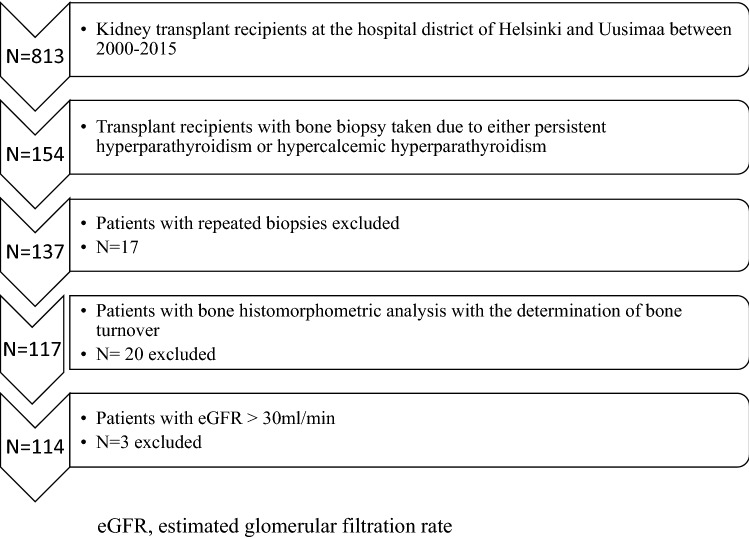
Flow chart of patient selection

A flow chart of the patients included in the study is presented in Fig. 1. Altogether 154 bone biopsies of 813 (19%) kidney transplant (deceased donors) recipients were taken. Repeat biopsies were performed for 17 patients and only the first biopsy was included in the statistical analysis. Bone turnover could be determined in 117 of the 137 patients and three patients with eGFR less than 30 ml/min/1.73 m^2^ were excluded. Thus, in total 114 patients were included in the statistical analysis.

Demographic characteristics of kidney transplant recipients are presented in Table 1. The median age, dialysis vintage, and the proportion of patients with diabetes were similar in both groups.

**Table 1:** Demographic and clinical characteristics of patients with high or normal/low turnover

	Median (IQR)	or *n* (%)		
Characteristic	All patients (*n* = 114)	High turnover in bone biopsy (group 1) (*n* = 80)	Normal or low turnover in bone biopsy (group 2) (*n* = 34)	*P*-value
Male	71 (61)	49 (61)	22 (65)	0.83
Age (years)	54 (45–62)	53 (46–62)	55 (44–64)	0.57
Previous KTX	10 (9)	9 (11)	1 (3)	0.28
Dialysis vintage (months) *n* = 112	24 (12–41)	27 (13–52)	19 (11–35)	0.07
Timing of bone biopsy after KTX (months)	28 (18–70)	24 (16–45)	64 (28–133)	<0.001
Indication for bone biopsy				
Elevated PTH level	40 (35)	24 (30)	16 (47)	
Hypercalcemia and elevated PTH level	74 (65)	56 (70)	18 (53)	
Diabetes mellitus	45 (39)	31 (39)	14 (41)	0.84
Coronary artery disease	19 (17)	13 (16)	6 (18)	1.0
Peripheral artery disease	14 (12)	9 (11)	5 (15)	0.76
Smoking *n* = 110	44 (39)	32 (41)	12 (35)	0.83
Previous PTX	10 (9)	5 (6)	5 (15)	0.16
PTX after biopsy	35 (31)	35 (44)	0	<0.001

*IQR* Interquartile range, *PTH* Parathyroid hormone, *KTX* Kidney transplantation, *PTX* Parathyroidectomy

Eighty patients (70%) presented with high bone turnover (group 1), and normal or low bone turnover (combined as group 2) was detected in 34 patients (25 patients with normal and 9 patients with low turnover), respectively. Twenty-four (60%) of 40 patients with normocalcemia and elevated PTH level and 56 (76%) of 74 patients with hypercalcemia (defined as iCa ≥ 1.3 mmol/L) combined with elevated PTH level had high -turnover bone disease.

The proportion of patients with the previous parathyroidectomy was higher in group 2, but the difference was not significant. Compared with group 1, bone biopsies in group 2 were taken 40 months later.

Of all patients, 91% were treated with glucocorticoids. Compared with the high-turnover group, the median cumulative dose of glucocorticoids was higher among patients with normal/low bone turnover (2851 mg vs. 4132 mg, p = 0.07). Calcium carbonate or acetate was administered to 21 (18%) patients. Active vitamin D supplements were administered to 31 (27%) patients, and seven (6%) patients were treated with cinacalcet at the time of biopsy. Bisphosphonates were administered to 39 (34%) patients. The proportion of patients treated with calcium and active vitamin D supplements, or with cinacalcet, did not differ between turnover groups (*p* = 0.430, *p* = 0.819, and *p* = 0.101, respectively). Compared with group 1, the proportion of patients treated with bisphosphonates was not significantly larger in group 2 (29% vs. 47%, *p* = 0.08).

### Bone Histomorphometric Parameters

Tetracycline labeling was found in 105 (92%) bone biopsies. BFR/BS and Ac. F could be determined in 87 (76%) and 60 (53%) patients, respectively. Either Ob.S/BS, Oc.S/BS, or BFR/BS, however, were available in all included patients. Bone histomorphometric parameters are shown in Table 2.

**Table 2:** Bone histomorphometric parameters according to turnover

Bone parameter	All biopsies (*n* = 114)	High turnover in bone biopsy (*n* = 80)	Normal or low turnover in bone biopsy (*n* = 34)	*P*-value
Bone formation rate/bone surface (µm^3^/µm^2^/year) *n* = 87	14.60 (6.60–31.22)	20.34 (10.95 −39.24)^a^	6.61 (3.65 −11.63)^b^	<0.001
Activation frequency (1/year) *n* = 60	0.46 (0.20–0.87)	0.64 (0.37–0.91)^c^	0.13 (0.10–0.35)^d^	<0.001
Osteoblastic surface/bone surface (%)	3.11 (1.38–6.70)	4.17 (2.0–8.79)	1.3 (0–2.92)	<0.001
Osteoclastic surface/bone surface (%)	1.19 (0–3.1.4)	1.94 (0.32–3.6)	0 (0–1.1)	<0.001
Osteoid surface/bone surface (%)	31.8 (21.0 −49.1)	36.2 (27.8–52.6)	19.1 (10.2–32.8)	<0.001
Osteoid thickness (µm)	8.3 (6.6–11)	10.0 (7.2–11.8)	6.75 (5.6–7.6)	<0.001
Mineralization lag time (days) *n* = 66	62.6 (41.3–99.4)	62.6 (42.4–92.8)^e^	77 (32.9–181.8)^f^	0.4
Bone volume/tissue volume (%)	20.1 (14.5–26.7)	20.5 (14.7–27.8)	17.4 (13.8–26.7)	0.55

^a^*n* = 68; ^b^*n* = 19; ^c^*n* = 48; ^d^*n* = 12; ^e^*n* = 52; ^f^*n* = 14

Mineralization was classified as abnormal/disturbed more frequently in patients with normal/low turnover compared to those with high bone turnover [14 (41%) patients vs. 10 (13%), *p* = 0.002].

Low bone volume was detected in 42 biopsies (37%). The prevalence of low bone volume did not differ between the turnover groups [28 (35%) in group 1 vs. 14 (41%) in group 2, *p* = 0.53].

### Biochemical Findings

Key laboratory values in high and normal/low bone turnover groups are shown in Table 3. The median eGFR did not differ between turnover groups [*p* = 0.93]. Accordingly, the median levels of iCa were similar between turnover groups [*p* = 0.05], but the levels of PTH were significantly higher in the high-turnover group compared to the normal/low turnover group [*p* = 0.007]. Phosphate levels were similar in both groups. ALP levels were significantly higher in the high-turnover group than in the normal/low turnover group [*p* = 0.001].

**Table 3:** Levels of bone mineral biomarkers at the time of bone biopsy according to bone turnover

		Median + (IQR)		
Variable (reference range)	All patients (*n* = 114)	High turnover in bone biopsy (group 1) (*n* = 80)	Normal or low turnover in bone biopsy (group 2) (*n* = 34)	*P*-value
crea µmol/L (60–100)	116 (94–132)	116 (90–135)	114 (95–130)	0.94
eGFR ml/min/1.73m^2^ (>89 ml/min)	56 (46–70)	55 (45–74)	57 (47–67)	0.93
iCa mmol/L per pH 7.4 (1.16–1.30)	1.32(1.27–1.37)	1.32 (1.28–1.38)	1.31 (1.24–1.36)	0.05
Pi mmol/L (0.71–1.53) *n* = 102	0.90 (0.72–1.05)	0.88 (0.72–1.05)	0.92 (0.75–1.05)	0.74
Pre-biopsy PTH ng/L (15–65)	125 (95–182)	140 (102–187)	107 (87–146)	0.007^*^
tALP U/L (35–105)	87 (67–129)	94 (74–142)	74 (60–89)	0.001^*^

*IQR* Interquartile range, *Crea* Creatinine, *eGFR* Estimated glomerular filtration rate, *iCa* Ionized calcium, *Pi* Phosphate, *PTH* Parathyroid hormone, *tALP* Total alkaline phosphatase

Conversion factors for units: plasma ionized calcium in mmol/L to mg/dL multiply by 4, inorganic phosphate in mmol/L to mg/dL multiply by 3.1, intact parathyroid hormone levels ng/L and pg/mL are equivalent

^*^Statistically significant

Sixteen (22%) of 74 patients with hypercalcemia and elevated PTH used either active vitamin D or calcium carbonate/acetate. Median iCa values in patients using these medications were 1.32 (IQR, 1.31–1.35) mmol/L compared to 1.37 (IQR, 1.33–1.40) mmol/L in patients without medications.

In a subanalysis, we compared the levels iCa, PTH, and ALP in patients using bisphosphonates (*n* = 39) and cinacalcet (*n* = 7) to those without these medications. Among bisphosphonates users with high and low/normal bone turnover (*n* = 23 and *n* = 16, respectively) the median levels of PTH and ALP did not differ from the levels of patients without these medications. The level of iCa was, however, lower in bisphosphonate users with low turnover compared to low turnover patients not using bisphosphonates (1.27 vs. 1.32 mmol/L, *p* = 0.04).

All patients using cinacalcet had high bone turnover. While the median levels of PTH and iCa were comparable to the levels of patients not using cinacalcet, the levels of ALP were significantly higher among cinacalcet users (92 vs. 195 U/L, *p* < 0.001).

### Logistic Regression Analysis

**Fig. 2:**
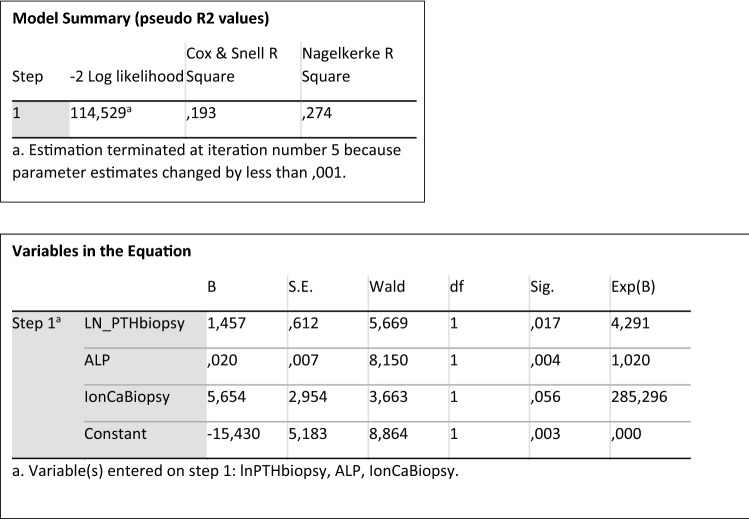
The specifics of the model

The logistic regression model (https://dev.arrak.fi/finne/ckd_mbd.html) constructed to predict high bone turnover disease (thus to answer the question “What is the probability that this transplant recipient has high bone turnover?”) included the variables iCa, ln PTH, and ALP. These variables were combined into one predicted probability of high-turnover disease. The risk score indicates the risk of having high bone turnover disease on bone biopsy based on the explanatory variables in the risk model. A risk score of, *e.g*., 0.69 thus equals to a 69% probability. This predicted probability was then used as a predictor for which usual diagnostic estimates, such as positive and negative predictive values, were calculated at various cutoffs of the predictor. The internal validation of the model was done with bootstrapping analysis. The specifics of the model are shown in Fig. 2.

When using a predicted probability cutoff of 80%, the positive predictive value was 91% (high turnover in bone biopsy was confirmed in 43 of 47 patients) and negative predictive value was 45%. At a predicted probability cutoff of 90%, the positive predictive value was 95% (high turnover in bone biopsy was confirmed in 20 of 21 patients) and negative predictive value was 35%.

The utility of predicted probability to predict high bone turnover was similar when patients using cinacalcet were excluded from analysis.

**Fig. 3:**
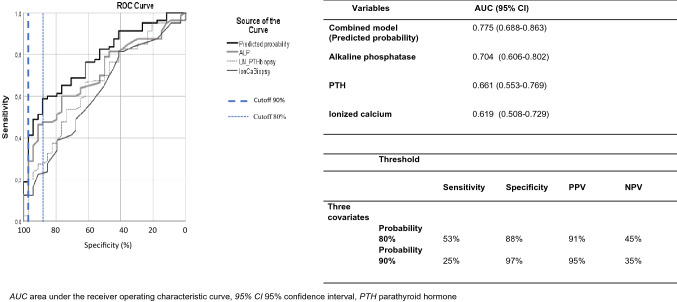
Diagnostic accuracy of mineral metabolism biomarkers and their combination for recognizing patients with high bone turnover

The model-derived probabilities were used to calculate AUCs for predicting high turnover. The AUC was 0.775 (95% CI 0.688–0.863) when using the logistic regression-based prediction, whereas it was 0.619 (95% CI 0.508–0.729), 0.661 (95% CI 0.553–0.769), and 0.704 (95% CI 0.605–0.800) for iCa, lnPTH, and ALP, respectively (Fig. 3).

In bootstrap analysis, the AUC of the prediction model was 0.756 (95% CI 0.600–0.874). The difference in AUC values between the prediction model and ALP was significant (*p* = 0.05).

### Parathyroidectomy

Thirty-five (44%) of 80 patients with bone biopsy-confirmed high bone turnover proceeded to subtotal parathyroidectomy. Parathyroidectomy was considered if bone turnover was notably increased with concurrent normal mineralization. Besides the bone biopsy finding, also the patient’s preferences for treatment guided the treating physician’s decision about parathyroidectomy. In the indication group with only elevated PTH levels, five (12%) patients proceeded to parathyroidectomy, while 30 (40%) patients with hypercalcemia and elevated PTH levels were operated. Among the 45 patients who did not proceed to parathyroidectomy, eight presented also abnormal mineralization, 34 had only mildly accelerated bone turnover, one refused the operation, and two were considered inoperable.

## Discussion

This retrospective study confirms the previous observations that high bone turnover does not normalize in approximately 10% of the patients after kidney transplantation [3–16]. It is clinically noteworthy, however, that while this study included solely patients with bone biopsies performed due to the clinical suspicion of high bone turnover, 24% of the patients with hypercalcemia combined with elevated PTH levels as well as 40% of the patients with normocalcemia in conjunction with elevated PTH levels had either normal or low bone turnover. This finding confirms the previous observation that the presence of hyperparathyroidism and hypercalcemia alone is not sufficient to diagnose high bone turnover [4]. The present study also aimed to determine the predictive value of mineral metabolism markers for the assessment of high bone turnover after kidney transplantation. Among included patients the presented logistic regression model recognized high bone turnover in a moderately accurate manner. If the access to the use of bone biopsy is limited, this model could serve as clinically available tool in recognizing high bone turnover in kidney transplant recipients with clinical suspicion of high bone turnover.

After kidney transplantation, PTH levels are approximately halved in the first six months, while thereafter the decrease is more gradual. Due to the long lifespan of parathyroid cells and the very slow involution of hyperplastic parathyroid glands after kidney transplantation, a significant proportion of kidney transplant recipients has persistently increased PTH levels [17]. The optimal PTH levels after transplantation thus remain unknown, and the definition of persistent hyperparathyroidism varies in the literature. Despite divergent time since transplantation, the prevalence of persistent hyperparathyroidism in this study extends the findings of previous retrospective studies done in the same era [12, 14]. In other studies, by contrast, the prevalence of persistent hyperparathyroidism was significantly higher [9, 11, 15]. Varying intervals since engraftment as well as differences in diagnostic criteria, medication, and immunosuppressive regimens probably account for this discrepancy. Interestingly, in this study, the median PTH level among patients with normal or low turnover was 107 ng/L, possibly suggesting that a higher *(i.e.,* 1.5 times the upper normal limit) PTH level may be the new normal set point after kidney transplantation. However, due to the limited number of bone histomorphometric studies in transplant recipients, the prevalence of post-transplantation high bone turnover is not well defined. Since all patients in our study were biopsied by an indication, the prevalence of post-transplantation high bone turnover is poorly comparable to other bone biopsy studies in contemporary cohorts [2, 4, 5, 8, 13, 16].

This study verifies the previous finding that persistent hyperparathyroidism is most prominent early after transplantation. The possibility of the drift from predominant persistent hyperparathyroidism to *de novo* secondary hyperparathyroidism, especially with decreasing graft function, must also be considered after transplantation. In this study, however, the graft function in both turnover groups was congruent and the time since transplantation was a significant negative predictor of high bone turnover. Since bone biopsies were taken at the late post-transplantation period, we assume, that bone turnover rate was in steady state. Age or diabetes did not correlate with bone turnover. The cumulative corticosteroid dose has previously been shown to decrease bone formation. In this study, the cumulative exposure to glucocorticoids was higher, although not significantly, among patients with normal/low bone turnover. This is, however, at least partially explained by the significantly later timing of bone biopsy in this group.

Although ALP, ln PTH, and iCa with AUCs 0.704, 0.661, and 0.619, respectively, performed better than the other parameters, they provided only suboptimal ability to predict high bone turnover. The logistic regression-based combination of these parameters, with an AUC of 0.775, improved the prediction of high turnover. It should be noted, however, that the AUC represents the predictive ability over the whole range of cutoff values of the predictors and therefore it is not very well suited for assessing the utility of the risk algorithm. In this study we showed that the risk algorithm was clinically useful for identifying patients with a significant risk of high-turnover disease when the cutoff values were high (>80%). The algorithm was especially beneficial if the predicted probability was higher than 90%. In this situation 95% of patients had high-turnover disease and thus, it could be argued that the bone biopsy could be omitted. In our study 18% of the patients had a predicted probability of higher than 90%. However, for patients with lower probabilities than 90% the algorithm was not similarly helpful. It should also be noted that the results of the presented algorithm apply only for the transplant recipients, who have received an engraftment over 18 months ago and have a high clinical suspicion of high bone turnover with comparable demographics and mineral metabolism markers.

The 4.3% incidence of post-transplantation parathyroidectomies in this study is within the range of reported rates from previous studies [39, 40]. At our institution, parathyroidectomy after kidney transplantation has been considered if the need for pharmacological treatment extends beyond one year or hypercalcemia persists despite the use of cinacalcet. In recent years, the number of parathyroidectomies after kidney transplantation has, however, decreased since most patients with high-turnover bone disease are treated before kidney transplantation.

The main strength of this study is its relatively large number of post-transplantation bone biopsy specimens taken due to the clinical suspicion of post-transplantation high bone turnover. A well-established policy at our institution preceding the parathyroidectomy is obtaining a bone biopsy to verify the diagnosis of high bone turnover. We thus assume that the prevalence of high bone turnover and the number of parathyroidectomies observed in this study adequately represent the kidney transplant recipients treated at our institution during the study period.

Several limitations of this study must also be addressed. A major limitation is the lack of an external validation due to the limited number of biopsies. The observational design of the study and single-center analysis potentially limits its generalizability. The use of Malluche´s reference values for BFR/BS and Ac. F may overestimate the prevalence of low turnover and underestimate the prevalence of high turnover. In this study, the diagnosis of high turnover was based also on other histomorphometric parameters, e.g., osteoblastic and osteoclastic surfaces. Although complete bone formation markers were not available for all bone samples, all biopsies were cross-checked by two experienced histomorphometrists (IB, HK) blinded to patients` other data, which increases the reliability of interpretation of bone histology. During the study period the levels of calcidiol and the prevalence of metabolic acidosis were not systemically evaluated in transplant recipients. In addition, information about the use of nutritional vitamin D and other immunosuppressive medication besides corticosteroid is lacking. Other potential confounders to this study are variations in immunosuppression, medications (including the introduction of cinacalcet in 2004), and transplant treatment protocols during the period of the study.

In conclusion, this study concurs with previous observations that hyperparathyroidism with or without hypercalcemia does not necessarily imply high bone turnover in kidney transplant recipients. However, the prediction of high bone turnover in the late engraftment period in patients with high clinical suspicion of bone disease can be improved by considering alkaline phosphatase levels in combination with iCa and PTH, as presented in the logistic regression model. In transplant recipients with a predicted probability score of less than 90%, bone biopsy verification is still needed if proceeding to parathyroidectomy is planned. It should also be noted that the presence of abnormal mineralization can only be excluded by the bone biopsy. As recently suggested by The European renal osteodystrophy initiative [41], the pooling of existing bone biopsy data from earlier studies would provide valuable information for further studies.

## Supplementary Information

Below is the link to the electronic supplementary material.

Supplementary file1 (DOCX 14 kb)

## Data Availability

Data cannot be shared publicly because of privacy of the patient participants, local legislation, and the New EU General data protection regulation (GDPR).
