# Supplementary material for: Clinical Prediction of High-Turnover Bone Disease After Kidney Transplantation
Source: Calcif Tissue Int. 2021 Oct 19;110(3):324–33. doi: 10.1007/s00223-021-00917-1 (PMC8860959; doi:10.1007/s00223-021-00917-1)
Supplement: Supplementary file 1 — Supplementary file1 (DOCX 14 kb) [file 223_2021_917_MOESM1_ESM.docx]

**Supplementary Information**

Supplementary material is available at <https://dev.arrak.fi/finne/ckd_mbd.html>.
